# Supplementary figures and images for: The effects on clinical trial activity of direct funding and taxation policy interventions made by government: A systematic review
Source: PLoS One. 2022 Sep 9;17(9):e0269021. doi: 10.1371/journal.pone.0269021 (PMC9462683; doi:10.1371/journal.pone.0269021)

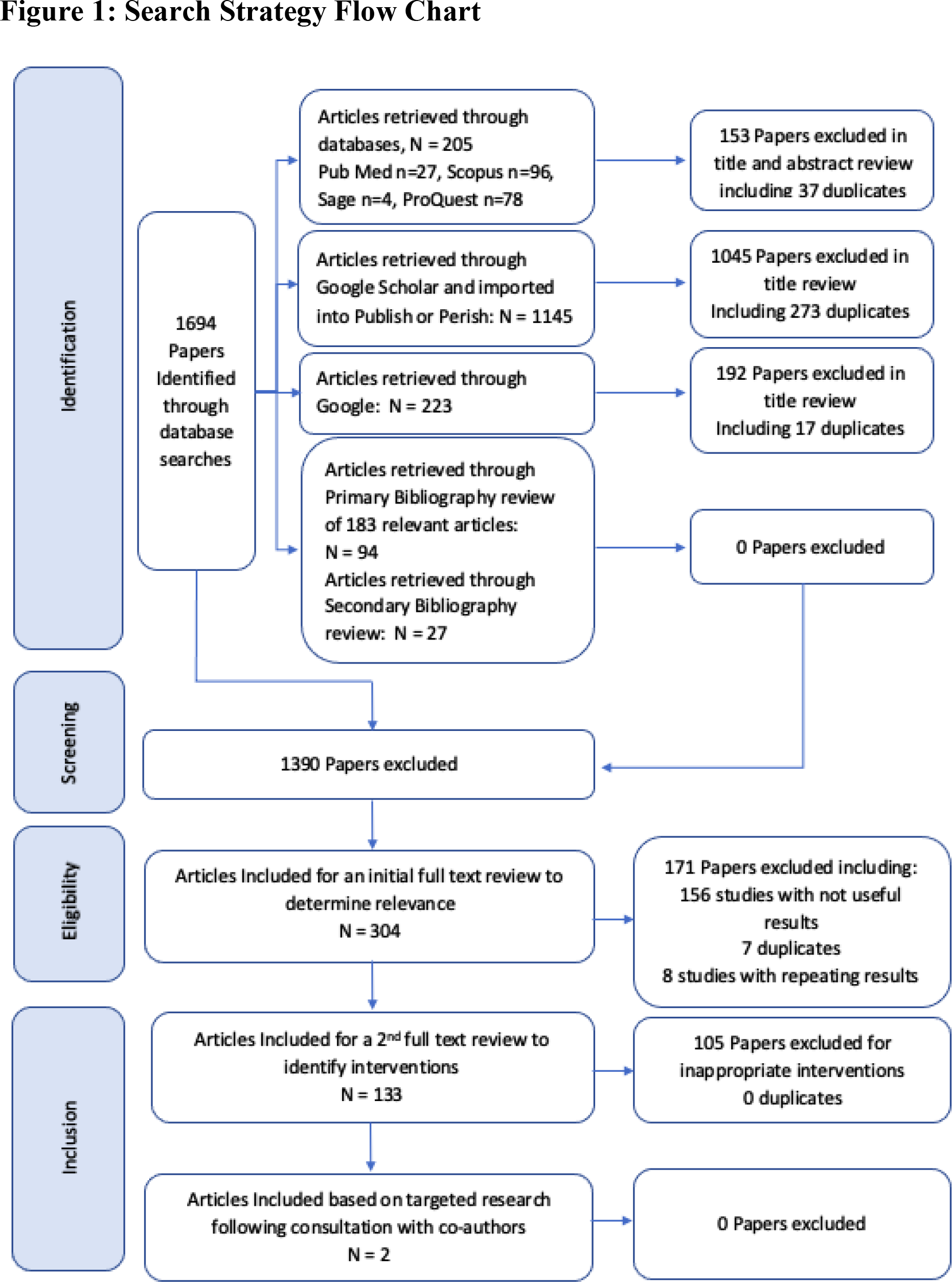

Supplement: S1 Fig — shows the search process that researchers SC and ER undertook. (TIF) [file pone.0269021.s002.tif]

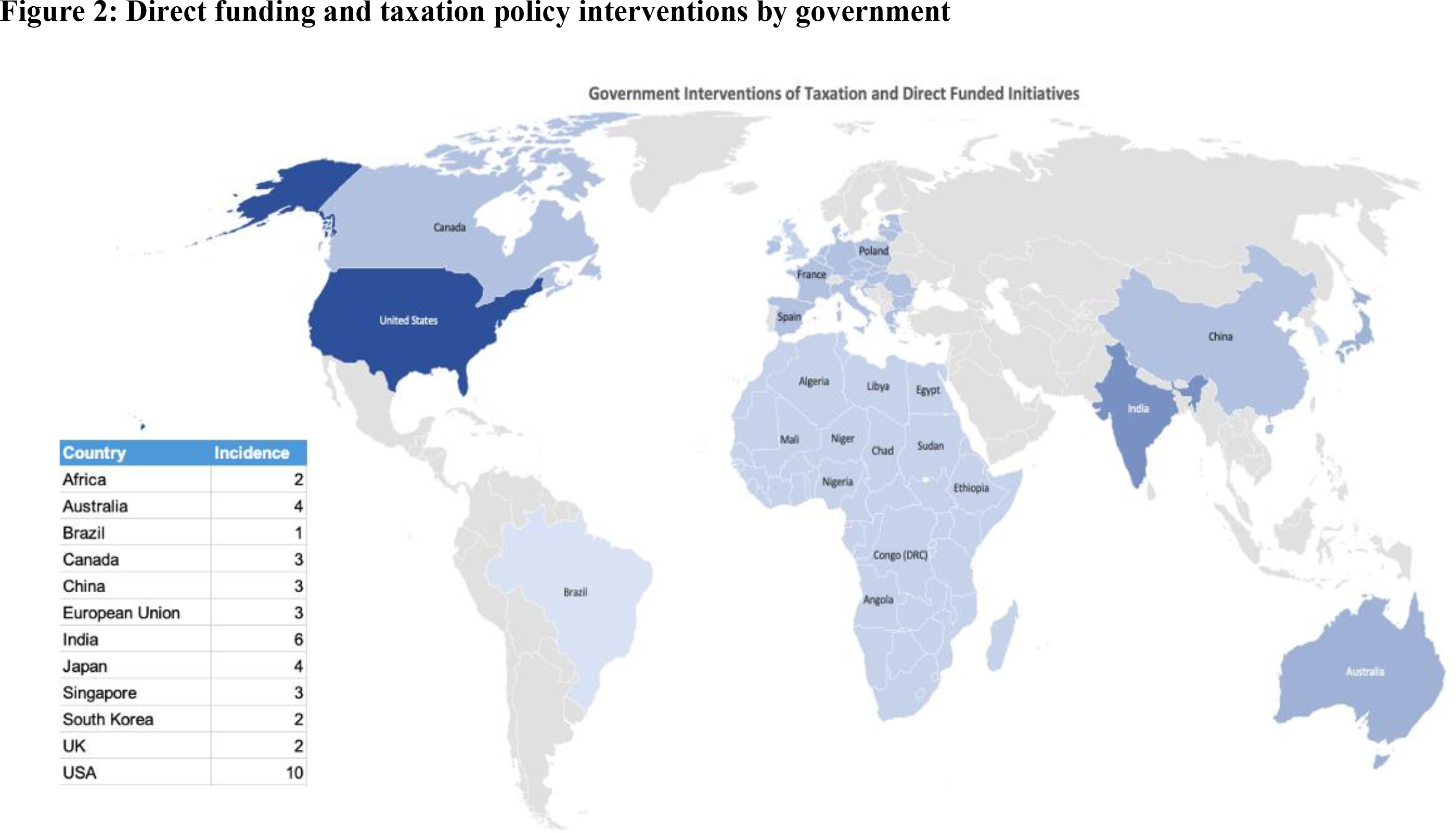

Supplement: S2 Fig — demonstrates the geographical locations that each intervention took place in. (TIF) [file pone.0269021.s003.tif]

**Appendix 1: Studies reporting direct funding initiatives or taxation policy interventions**


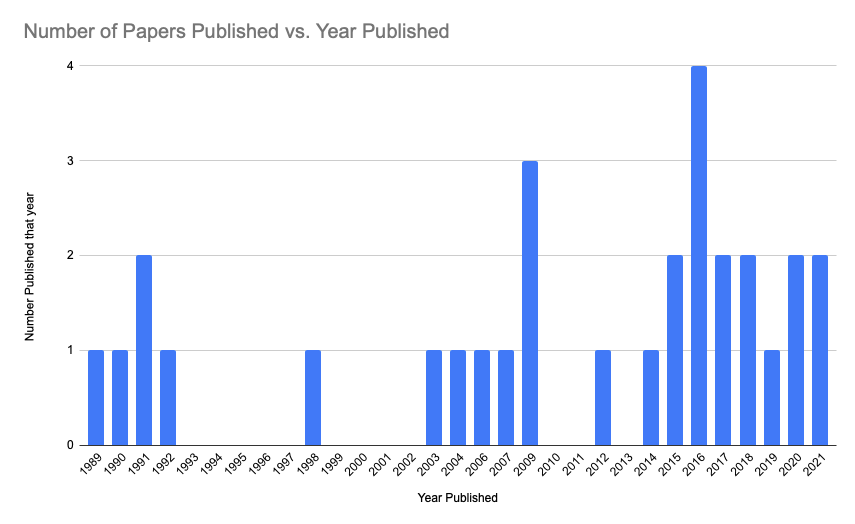

Supplement: S1 Appendix — Graph shows the year that each identified paper was published. (DOCX) [file pone.0269021.s004.docx]
