# Supplementary material for: The effects on clinical trial activity of direct funding and taxation policy interventions made by government: A systematic review
Source: PLoS One. 2022 Sep 9;17(9):e0269021. doi: 10.1371/journal.pone.0269021 (PMC9462683; doi:10.1371/journal.pone.0269021)
Supplement: Attachment — Submitted filename: Response to Reviewers.docx [file pone.0269021.s005.docx]

21^st^ August 2022

RE: PONE-D-22-13387

Dear Editors,

Thank you so much for your comments and suggestions, as well as those of the reviewers.

Please see below a point by point response to each comment raised by yourselves and the reviewers.

We have numbered the requested changes and formatted them in italics, while the response is marked below each item in bold.

Happy to make any other changes you believe it needs.

Many thanks in advance

Sam

1. *Please ensure that your manuscript meets PLOS ONE's style requirements, including those for file naming. The PLOS ONE style templates can be found at
   https://journals.plos.org/plosone/s/file?id=wjVg/PLOSOne_formatting_sample_main_body.pdf and* 
   [*https://journals.plos.org/plosone/s/file?id=ba62/PLOSOne_formatting_sample_title_authors_affiliations.pdf*](https://journals.plos.org/plosone/s/file?id=ba62/PLOSOne_formatting_sample_title_authors_affiliations.pdf).

**Response:	Completed**

2. *We note that Figure 2 in your submission contain map/satellite images which may be copyrighted. All PLOS content is published under the Creative Commons Attribution License (CC BY 4.0), which means that the manuscript, images, and Supporting Information files will be freely available online, and any third party is permitted to access, download, copy, distribute, and use these materials in any way, even commercially, with proper attribution. For these reasons, we cannot publish previously copyrighted maps or satellite images created using proprietary data, such as Google software (Google Maps, Street View, and Earth). For more information, see our copyright guidelines:* [*http://journals.plos.org/plosone/s/licenses-and-copyright*](http://journals.plos.org/plosone/s/licenses-and-copyright)*.
    
   We require you to either (1) present written permission from the copyright holder to publish these figures specifically under the CC BY 4.0 license, or (2) remove the figures from your submission:
    
   a) You may seek permission from the original copyright holder of Figure 2 to publish the content specifically under the CC BY 4.0 license.  
    
   We recommend that you contact the original copyright holder with the Content Permission Form (http://journals.plos.org/plosone/s/file?id=7c09/content-permission-form.pdf) and the following text:
   “I request permission for the open-access journal PLOS ONE to publish XXX under the Creative Commons Attribution License (CCAL) CC BY 4.0 (http://creativecommons.org/licenses/by/4.0/). Please be aware that this license allows unrestricted use and distribution, even commercially, by third parties. Please reply and provide explicit written permission to publish XXX under a CC BY license and complete the attached form.”
    
   Please upload the completed Content Permission Form or other proof of granted permissions as an "Other" file with your submission.
    
   In the figure caption of the copyrighted figure, please include the following text: “Reprinted from [ref] under a CC BY license, with permission from [name of publisher], original copyright [original copyright year].”
    
   b) If you are unable to obtain permission from the original copyright holder to publish these figures under the CC BY 4.0 license or if the copyright holder’s requirements are incompatible with the CC BY 4.0 license, please either i) remove the figure or ii) supply a replacement figure that complies with the CC BY 4.0 license. Please check copyright information on all replacement figures and update the figure caption with source information. If applicable, please specify in the figure caption text when a figure is similar but not identical to the original image and is therefore for illustrative purposes only.
   The following resources for replacing copyrighted map figures may be helpful:
    
   USGS National Map Viewer (public domain): http://viewer.nationalmap.gov/viewer/
   The Gateway to Astronaut Photography of Earth (public domain): http://eol.jsc.nasa.gov/sseop/clickmap/
   Maps at the CIA (public domain): https://www.cia.gov/library/publications/the-world-factbook/index.html and https://www.cia.gov/library/publications/cia-maps-publications/index.html
   NASA Earth Observatory (public domain): http://earthobservatory.nasa.gov/
   Landsat: http://landsat.visibleearth.nasa.gov/
   USGS EROS (Earth Resources Observatory and Science (EROS) Center) (public domain): http://eros.usgs.gov/#
   Natural Earth (public domain):* [*http://www.naturalearthdata.com/*](http://www.naturalearthdata.com/)

**Response:	Figure 2 was created using the map tool on a spreadsheet on a fully licenced version of Microsoft excel, (as was appendix 1). As such it complies with Microsoft’s copyright permissions: “You may sell a spreadsheet, database, or PowerPoint deck you made using Microsoft software. The spreadsheet, database, or PowerPoint deck must be created using legitimate, licensed Microsoft software.” Please see:** [**https://www.microsoft.com/en-us/legal/intellectualproperty/copyright/permissions**](https://www.microsoft.com/en-us/legal/intellectualproperty/copyright/permissions)

3. *We note you have included tables to which you do not refer in the text of your manuscript. Please ensure that you refer to Tables 2 and 3 in your text; if accepted, production will need this reference to link the reader to the Table.*

**Response:	Thank you. This has been amended. Please see the bottom of page 6. The heading has been amended as follows: “Characteristics of the interventions and the available outcome data (Table 2 & 3)” and at the bottom of that section, the following sentence has now been inserted: “Characteristics of the interventions for which outcome data was available is outlined in Table 2, while the characteristics of the interventions for which no outcome data was available is outlined in Table 3.”**

4. *Please upload a copy of Figure 3, to which you refer in your text on page xx. If the figure is no longer to be included as part of the submission please remove all reference to it within the text.*

**Response: Apologies, all references to Figure 3 have now been deleted.**

5. *Please review your reference list to ensure that it is complete and correct. If you have cited papers that have been retracted, please include the rationale for doing so in the manuscript text, or remove these references and replace them with relevant current references. Any changes to the reference list should be mentioned in the rebuttal letter that accompanies your revised manuscript. If you need to cite a retracted article, indicate the article’s retracted status in the References list and also include a citation and full reference for the retraction notice.*

**Response:	The reference list has been reviewed and substantial changes made to the formatting throughout to keep in line with PLOS ONE’s publishing style.**

Reviewer Two Noted:

6. *According to my opinion article is focused on very interesting and actual topics. I really appreciate the quality of the research and also very interesting conclusions. In the text, I found a few grammatical mistakes. The authors used a lot of quality sources. Some of them are older. I would suggest that authors could try to find new sources.
   In general, I recommend publishing a paper.*

***Response 1: The paper has been reviewed for grammatical mistakes and minor revisions made.***

***Response 2: All references have been reviewed. The older references that are referred to were captured in the search processes documented in the paper. Removing these references would require structural changes to the search and would alter the paper’s findings. All other references – not found in the systematic review – are current.***
